# The combination of complex karyotype subtypes and IGHV mutational status identifies new prognostic and predictive groups in chronic lymphocytic leukaemia

**DOI:** 10.1038/s41416-019-0502-x

**Published:** 2019-06-18

**Authors:** Andrea Visentin, Laura Bonaldi, Gian Matteo Rigolin, Francesca Romana Mauro, Annalisa Martines, Federica Frezzato, Silvia Imbergamo, Edoardo Scomazzon, Stefano Pravato, Maria Antonella Bardi, Maurizio Cavallari, Eleonora Volta, Francesco Cavazzini, Maurizio Nanni, Ilaria Del Giudice, Monica Facco, Anna Guarini, Gianpietro Semenzato, Robin Foà, Antonio Cuneo, Livio Trentin

**Affiliations:** 1 Hematology and Clinical Immunology Unit, Department of Medicine, University of Padua, Padua, Italy; 2 Venetian Institute of Molecular Medicine, Padua, Italy; 3 Immunology and Molecular Oncology Unit, Veneto Institute of Oncology IOV-IRCSS, Padua, Italy; 4 Hematology section, Department of Medical Sciences, Azienda Ospedaliera-Universitaria, Arcispedale S. Anna, University of Ferrara, Ferrara, Italy; 5 Hematology division, Department of Translational and Precision Medicine, “Sapienza” University, Rome, Italy

**Keywords:** Chronic lymphocytic leukaemia, Prognostic markers, Predictive markers

## Abstract

**Background:**

Complex karyotype (CK) is a heterogeneous category with a negative impact in chronic lymphocytic leukaemia (CLL). Our group has recently reported that CK patients with major structural abnormalities (i.e. CK2) are characterised by a worse prognosis, as compared to other lesions within CK(CK1).

**Methods:**

We performed a multicentre retrospective study to test whether the combination of CK subtypes with *IGHV* status could be a relevant prognostic and predictive tool.

**Results:**

Among 522 patients 13% harboured CK2, 41% CK1 and/or U-IGHV (U-CK1) and 46% M-IGHV without any CK subtypes (M-noCK). After a median follow-up of 5.8 years, CK2 patients had the shortest TTFT (5-year TTFT 31%, 39 and 81%, *p* < 0.0001) and OS (5-year OS 67%, 85 and 93%, *p* < 0.0001) as compared to U-CK1 or M-noCK cases, regardless of TP53 abnormalities. CK2 patients also had the worst outcome after chemoimmunotherapy. In fact, the median TTNT after FCR or BR was 1.86 and 4.79 years for CK2 and U-CK1, but not reached for M-noCK patients (*p* < 0.0005).

**Conclusions:**

We herein suggest that the combined assessment of the *IGHV* mutational status and CK subtypes refines the prognostication of CLL, allowing to identify M-IGHV patients without any CK subtypes who are characterised by an indolent disease and excellent outcome after chemoimmunotherapy.

## Background

Chronic lymphocytic leukaemia (CLL), the most common leukaemia in western countries, is a remarkably heterogeneous disease, with some patients never requiring treatment and others with a highly aggressive and/or rapidly progressive clinical course.^1,2^ The three most important CLL prognostic factors, variables capable of defining subjects at a higher risk of progression or death, and predictive genetic markers, which identify subjects who will relapsed earlier after treatments, are represented by leukemic cell cytogenetic assessed by fluorescence in situ hybridisation (FISH), *TP53* abnormalities, including mutations and deletions, and the mutational status of the variable region of the immunoglobulin heavy chain (*IGHV*) genes.^3^

Recent studies have shown that current FISH analysis, according to Dohner’s hierarchical model, underestimates the true genetic complexity revealed by chromosome banding analysis. In fact, 22–36% of CLL cases with “normal” FISH carry chromosomal aberration at karyotype. In particular, complex karyotype (CK), defined by the presence of at least three chromosome lesions in the same clone, is detectable in 14–34% of CLL cases^4–7^ and is emerging as a new negative prognostic biomarker associated with an adverse outcome^4,8^ and worse response to chemoimmunotherapy^5,9^ as well as to novel agents,^10,11^ regardless of the CLL-IPI score, unmutated *IGHV* genes and 11q/17p deletions.^6^ However, the CK itself is a heterogeneous quantitative and qualitative cytogenetic category that includes numerical (i.e. monosomies and trisomies) and structural abnormalities (i.e. balanced and unbalanced translocations, marker chromosomes, isochromosomes, deletions, insertions and additions). Rigolin et al. recently demonstrated that among 90 CK cases the presence of major structural abnormalities at CLL diagnosis identifies a subset of patients with a poor outcome and distinct mRNA expression profile.^12^ However, it is unknown whether the prognostic strength of CK could be improved when combined with a stable marker such as the IGHV mutational status, and whether this approach could help to identify patients who can gain the maximum benefit from chemoimmunotherapy.

In this multicentre retrospective study, we demonstrated that in 522 CLL patients the combination of CK subtypes and *IGHV* status provides relevant prognostic data, allowing to refine the prognostic stratification of CLL patients, and to identify M-IGHV patients without any CK subtypes who are characterised by an indolent disease and excellent outcome after chemoimmunotherapy.

## Methods

### Study design

Inclusion criteria for this study were diagnosis of CLL according to the 2008 iwCLL criteria,^13^ age >18 years and chromosome banding analysis performed within 1 year from diagnosis. Data included in the comparative analysis were gender, age, Binet stage,^13^ need for chemotherapy, CD38 expression (performed as previously reported^2^ with a cut-off value of 30%), cytogenetics detected by fluorescence in situ hybridisation (FISH),^14^ *IGHV* mutational analysis^15^ and *TP53* abnormalities including gene deletions or mutations.^16^ The primary endpoint was the impact of the combination of CK subtypes with *IGHV* status on the overall survival (OS) of patients. The correlation with clinico-biological variables and its impact on time to first treatment (TTFT) and relapse after chemoimmunotherapy were considered as secondary endpoints. This study was approved by the local research ethics committee and informed consent was obtained from all patients.

### Chromosome banding analysis

Cytogenetic analysis was performed on peripheral blood after a 72 h exposure of 500 µM CpG ODN DSP30 (Roche, Risch, CH) mitogen + 20 U/mL IL2 (Roche). Cultures were exposed overnight to 0.1 µg/mL colcemid (Gibco^®^ Karyomax Colcemid, ThermoFisher, Waltham, MA USA) to obtain metaphases and then they were harvested following standard procedures. Karyotype was described after the analysis of at least 25 G-banded metaphases using the IKAROS software (MetasYstems, Altlhusseim, Germany), according to International guidelines (ISCN 2016). Complex karyotype (CK) was defined by the presence of three or more chromosome abnormalities in the same clone.^4,6,17,18^ Moreover, based on the type of chromosome changes among CK, we termed Type-2 CK (CK2) those cases with major structural rearrangements (i.e. unbalanced translocations, chromosomes addition, insertion, duplications, ring, dicentric and marker chromosomes).^12^ Whereas, complex karyotypes with balanced translocations, deletions, monosomies or trisomies were called as type 1 (CK1).^12^

### IGHV mutational status

Analysis of the IGHV mutational status was performed within 12 months from diagnosis on peripheral blood CLL cells from fresh samples or frozen purified CLL cells harvested in DMSO. RNA was extracted from 2 × 10^6^ B cells using the RNeasy™ Total RNA kit (Qiagen, Hilgen, Germany) and reverse transcribed using the SuperScript™ Preamplification System for first-strand cDNA synthesis (Life Technologies, Carlsbad, CA). The CLL B-cell HV gene family was assigned as previously described.^19,20^ HV gene sequences were determined by amplifying 5 μl of the original cDNA using the appropriate HV leader and HC primers. PCR products were sequenced directly after purification with Wizard PCR Preps (Promega, Madison, WI) using an automated genetic analyser (3130 ABI Applied Biosystems, Foster City, CA, USA). Sequences were analysed using the IMGT/VQUEST and BLAST softwares^21^ to detect VDJ junction. Sequences homology <98, from the corresponding germline gene, were considered mutated (M-IGHV), as opposite to unmutated (U-IGHV) cases.^19,22^

### Cytogenetic by fluorescence in situ hybridisation (FISH) and TP53 mutations

FISH was performed on standard cytogenetic preparations from peripheral blood.^20^ The slides were hybridised with the multicolour probe sets LSI p53/LSI ATM and LSI D13S319/LSI 13q34/ CEP12 (Vysis-Abbott, Des Plaines, IL, USA), according to the manufacturer’s protocol.^23^ Three hundred interphase nuclei were analysed for each probe and the cut-off for positive values were 10% for deletion of 11q22.3 (ATM), 13q14.3 (D13S319) and 17p13.1 (TP53) loci and 5% for trisomy 12. High-risk FISH refers to 11q- or 17p-. As opposite, Low-risk FISH included 13q14 deletion or normal FISH. Patients harbouring trisomy 12 were considered at intermediate risk. *TP53* gene sequencing between and analysis were performed according to ERIC guidelines.^16^

### Treatment

Patients were treated accordingly to the 2008 iwCLL guidelines.^13^ Fludarabine or bendamustine-based regimens, with or without rituximab were used as first-line treatment in fit patients; chlorambucil with or without antiCD20 monoclonal antibody was used in elderly and/or unfit patients. FCR (fludarabine, cyclophosphamide and rituximab)^24^ and BR were administrated at standard doses.^25^ Since January 2015 patients with *TP53* abnormalities were treated with BCR inhibitors, ibrutinib or idelalisib plus rituximab.

### Statistical analysis

Categorical variables were compared by Chi-square test (for Binet stages and FISH) or Fisher exact’s test (gender, treatment, CD38, *TP53* and *IGHV*), when appropriate. Continuous variables (median age) were compared with Mann–Whitney test. TTFT was calculated starting from the date of diagnosis to treatment (event) or last known follow-up (censored).^13^ Time to next treatment (TTNT) was calculated as months from first-line FCR or BR to subsequent therapy or last known follow-up. OS were calculated starting from the date of diagnosis to death for any cause, or last known follow-up. Survival analyses were performed by the Kaplan–Meier method and the Log-rank test was used to compare survival curves between groups. Cox regression model was employed to estimate hazard ratios (HR). The cox proportional hazard assumption was assessed based on the scaled Schoenfeld residuals. The stability of our model was internally validated by bootstrap .632 method with B = 522. The Harrell concordance index (c-index; 1.0 indicates perfect discrimination while a value 0.5 indicates equivalence to chance) was used to compare our prognostic model with Dohner’s,^23^ FISH-IGHV and CLL-IPI models.^26^ The prediction error was calculated as 1—c-index, corrected for optimism and estimated by .632 bootstrap method. A *p* value >0.05 was considered as not significant. Statistical analysis was performed with R (an open source statistical package downloadable from http://www.r-project.org).

## Results

### Patients’ characteristics

We gathered data from 522 CLL patients with chromosome banding analysis and *IGHV* status assessed within 12 months from diagnosis (Table 1). The median age at diagnosis of the whole population was 65 years, 61% were males, 76% at Binet A stage, the median β2-microglobulin was 3.27 mg/L, 47% U-IGHV, 9% patients harboured *TP53* abnormalities and 19% a CK. Two hundred and thirty-two patients received at least one line of therapy (31% FCR, 16% BR, 8% ibrutinib, 5% chlorambucil-antiCD20, 40% other treatments such as FC or chlorambucil alone, etc.) and 80 died over a median follow-up of 5.8 years. According to the subtype of CK, 30 (30%) showed a CK1 and 69 (70%) a CK2. In this latter group 35% were M-IGHV and 65% were non-mutated conformation of IGHV gene (Table 1).

**Table 1:** Clinical and biological features of patients

	Population (*n* = 522)	M-noCK (*n* = 240)	U-CK1 (*n* = 213)	CK2 (*n* = 69)	*p* values
Gender					
Female	203 (39%)	88 (37%)	87 (41%)	28 (41%)	0.6298
Male	319 (61%)	152 (63%)	126 (59%)	41 (59%)	
Age (years)					
Median ± sd	65 ± 10	59 ± 11	65 ± 12	70 ± 10	0.0053
Binet stage					
A	396 (76%)	164 (69%)	189 (89%)	43 (62%)	<0.0001
B	91 (17%)	66 (27%)	8 (4%)	17 (25%)	
C	35 (7%)	10 (4%)	16 (7%)	9 (13%)	
β2-microglobulin (mg/L)					
Median ± sd	2.92 ± 1.53	2.47 ± 1.55	3.17 ± 1.34	3.27 ± 1.78	<0.0001
CD38^a^					
<30%	388 (74%)	207 (88%)	140 (67%)	41 (62%)	<0.0001
≥30%	121 (23%)	27 (12%)	69 (33%)	25 (38%)	
IGHV status					
M-IGHV	279 (53%)	240 (100%)	15 (7%)	24 (35%)	n.a.
U-IGHV	243 (47%)	0 (0%)	198 (93%)	45 (65%)	
FISH^a^					
Normal	153 (29%)	86 (38%)	59 (28%)	8 (12%)	<0.0001
13q	196 (38%)	122 (54%)	56 (27%)	18 (26%)	
+12	76 (15%)	17 (8%)	52 (25%)	7 (10%)	
11q	50 (10%)	4 (2%)	33 (16%)	13 (19%)	
17p	36 (7%)	3 (1%)	10 (5%)	23 (33%)	
TP53^a^					
Normal	469 (90%)	229 (97%)	197 (92%)	43 (62%)	<0.0001
Abnormal	48 (9%)	6 (3%)	16 (8%)	26 (38%)	
N. Chr. abn.					
0	167 (32%)	117 (49%)	50 (24%)	0 (0%)	<0.0001
1–2	258 (49%)	123 (51%)	135 (63%)	0 (0%)	
3–4	53 (10%)	0 (0%)	25 (12%)	28 (41%)	
≥5	44 (9%)	0 (0%)	3 (1%)	41 (59%)	

*sd* standard deviation, *M-IGHV* mutated IGHV gene, *U-IGHV* unmutated IGHV gene, *N. Chr. abn.* number of chromosomal abnormalities, *CK* complex karyotype, *M-noCK* M-IGHV without CK, *U-CK1* U-IGHV and/or type 1 CK, *CK2* type 2 CK. *n.a.* not applicable

^a^Missing data  =  3% about CD38 expression, 1% cytogenetic by FISH and 1% for TP53 abnormalities (including deletions and/or mutations)

As a preliminary step for our further analysis, we confirmed the established prognostic role of U-IGHV, CK and CK with major unbalanced abnormalities (i.e. CK2) in our dataset (Fig. S1A-F). The 10-year OS was 60% in U-IGHV and 89% M-IGHV group (*p* < 0.0001, Fig. S1D); 58% for CK and 79% for no-CK patients, respectively (*p* < 0.0001, Fig. S1E); 49% vs 66% vs 79% for CK2, CK1 and no-CK (*p* < 0.0001, Fig. S1F), respectively. Due to the superimposable trend and absence of any statistical difference between OS curves of the CK1 and U-IGHV groups, these patients were grouped and analysed together (U-CK1), as well as M-IGHV and no-CK patients (M-noCK) (Figure S1G). Sixty-nine (13%) patients of the whole population harboured CK2, 213 (41%) CK1 or U-IGHV (U-CK1) and 240 (46%) M-IGHV without any subtype of CK (M-noCK). The former group was characterised by a more advanced stage at diagnosis (Binet C, 13% vs 7% vs 4%, *p* < 0.0001), higher levels of β2-microglobulin (2.47 mg/L vs 3.17 mg/L vs 3.27 mg/L, *p* < 0.0001), lower number of cases with low-risk FISH (i.e. 13q- or normal FISH, 38% vs 55% vs 92%, *p* < 0.0001), but an increased prevalence of *TP53* aberrations (38% vs 8% vs 3%, *p* < 0.0001) and number of chromosomal abnormalities (≥5 lesions, 0% vs 1% vs 59%), as compared to the other two groups (Table 1).

### Prognostic impact of CK subtypes and IGHV combination

**Fig. 1:**
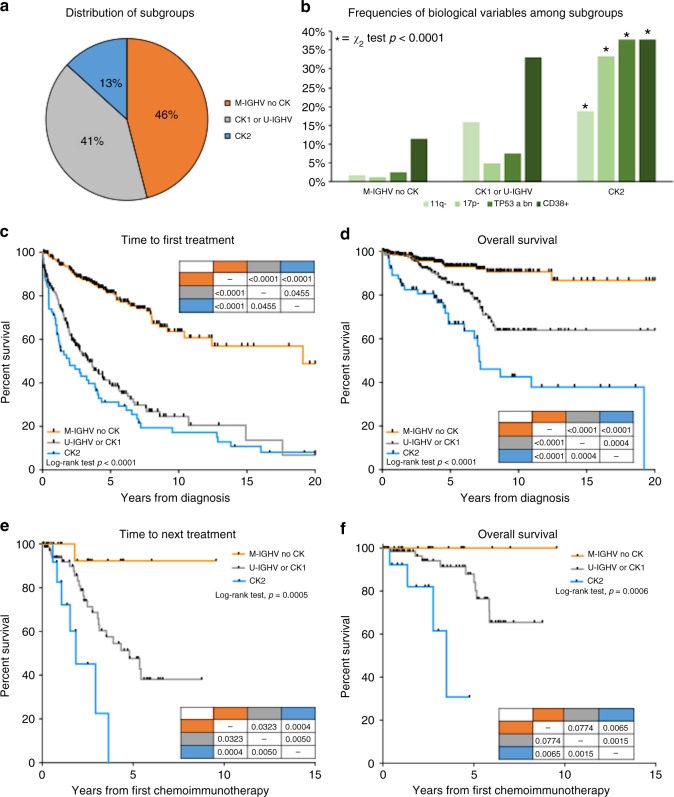
In the upper-left panel **a** the apple-pie graph represents the distribution of patients harbouring type 2 complex karyotype (CK2, 13%), type 1 CK or unmutated *IGHV* gene (U-CK1, 41%) and mutated IGHV gene without any CK subtypes (M-noCK, 46%). The upper-right panel **b** shows histograms for frequencies of biological variables among identified subgroups. CK2 subgroups was significantly enriched for CD38 ≥ 30% (CD38+), 11q deletion (11q−), 17p deletions (17p−), *TP53* abnormalities (*TP53* abn) cases compared to M-noCK or U-CK1 subjects. Panels **c**–**f** show Kaplan–Meier curves for survival analysis. Patients with CK2 (light grey curve) had the shortest time to first treatment (**c**) and overall survival (**d**) from diagnosis, as well as the worst time to next treatment (**e**) and overall survival (**f**) after chemoimmunotherapy with FCR or BR as compared with U-CK1 (dark grey curves) and M-noCK cases (black curves)

We observed that patients with CK2 have a significantly shorter TTFT and OS compared to U-CK1 or M-noCK cases (Fig. 1c, d). The median OS was 7.1 years for CK2 patients but was not reached for U-CK1 and M-noCK patients (*p* < 0.0001). The 5-year OS was 67%, 85%, and 93% for CK2, U-CK1 and M-noCK cases, respectively (Fig. 1d, *p* < 0.0001). The median and 5-year TTFT were 1.97 vs 3.40 vs 19.1 and 31% vs 39% vs 81% for CK2, U-CK1 and M-noCK cases (Fig. 1c, *p* < 0.0001), respectively. For both TTFT and OS, each curve was statistically different from the all others (Fig. 1c, d). Moreover, the worse prognosis of CK2 patients was independent of the *TP53* (Fig. S2A-B, *p* = 0.0845 and 0.8122 for TTFT and OS) and *IGHV* status (Fig. S2C-D, *p* = 0.0755 and 0.2230 for TTFT and OS). In particular, multivariate analysis confirmed that U-CK1 patients have a 3-fold higher risk of starting treatment and of dying than M-noCK patients (Table 2, *p* < 0.0001). Similarly, CK2 patients have 5- and 7-fold risk of undergoing first-line therapy and of dying compared to M-noCK subjects (Table 2, *p* < 0.0001). Other variables associated with shorter TTFT and OS at univariate and multivariate analysis were advanced Binet stage, CD38+, U-IGHV, 11q−, 17p− and *TP53* abnormalities (Table S1-S2). Age >65 years at diagnosis also predicted a shorted OS at multivariate analysis (Table S2).

**Table 2:** Hazard ratios (HR) for the combination of IGHV mutational status with CK subtypes

	Univariate analysis			Multivariate analysis		
	HR	95% C.I	*p* values	HR	95% C.I	*p* values
TTFT						
M-noCK	1.00	–	–	1.00	–	–
U-CK1	4.31	3.14–5.90	<0.0001	3.98	2.87–5.52	<0.0001
CK2	4.89	2.99–7.99	<0.0001	5.12	3.5–7.47	<0.0001
OS						
M-noCK	1.00	–	–	1.00	–	–
U-CK1	3.10	1.81–5.30	<0.0001	3.14	1.75–5.64	0.0001
CK2	7.07	3.13–15.08	<0.0001	7.37	3.97–13.69	<0.0001

*95% C.I.* 95% confidential interval, *M-noCK* mutated IGHV without complex karyotype, *U-CK1* unmutated IGHV and/or type 1 complex karyotype, *CK2* type 2 complex karyotype, *TTFT* time to first treatment, *OS* overall survival

Our model was also internally validated by bootstrap .632 method, showing a prediction error of 0.31 and 0.34 for TTFT and OS, respectively. Finally, the c-indexes for the FISH hierarchical model were 0.64 and 0.61 (Fig. S3A-B), the IGHV-FISH system 0.69 and 0.63 (Fig. S3C-D), CLL-IPI 0.67 and 0.62 (Fig. S3E-F) while for our proposed model they were 0.70 and 0.69 for TTFT and OS, respectively. These results indicate that our model was slightly better than other commonly used prognostic scores applied to our population.

### Predictive impact of CK subtypes and IGHV combination

The combination of the IGHV status and CK subtypes also provides predictive information after first-line therapy (*n* = 160 patients, *p* < 0.0001 for both TTNT and OS, Fig. S2G-H). In particular, focusing on 107 patients treated with FCR or BR, 20% were M-noCK, 67% U-CK1 and 13% CK2. We observed that only one of the M-noCK cases relapsed and that no patient has died after a median follow-up of 43 months as compared with the other two subgroups (Fig. 1e, f). The median TTNT was 1.86 and 4.79 years for CK2 and U-CK1, but not reached for M-noCK patients (*p* < 0.0005, Fig. 1e). The estimated 3-year TTNT was 92%, 69 and 23%, for M-noCK, U-CK1 and CK2 patients (*p* < 0.0005, Fig. 1e), respectively. The median OS was 3.5 years for CK2 but not reached for both U-CK1 and M-noCK cases (*p* = 0.0006, Fig. 1f). The 3-year OS was 100%, 94 and 62% for M-noCK, U-CK1 and CK2 patients (*p* = 0.0006, Fig. 1f), respectively. Variables associated with TTNT and OS at univariate and multivariate analysis were reported in Table S3 and S4. CK2 predicted a shorter TTNT and OS also at multivariate analysis (*p* = 0.0055 and *p* = 0.0113, respectively).

Subsequently, we compared CK2 patients treated with FCR or BR (*n* = 14) with those who received ibrutinib first-line (*n* = 7). Although the 3-year TTFT and OS for patients treated with chemoimmunotherapy and those with ibrutinib were 67% vs 22% and 100% vs 62%, respectively, these differences were not statistically significant (*p* = 0.2479 for TTNT and *p* = 0.2011 for OS, Fig. S4A-B).

## Discussion

Chromosome banding analysis in CLL is capable of identifying chromosomal abnormalities that are missed by FISH analysis, sometimes fulfilling the criteria of CK.^4,18,27,28^ In this retrospective study we confirmed in a large cohort of patients that CK is not a single entity but is a quantitative and qualitative cytogenetic heterogeneous category. CK patients with major structural lesions (i.e. CK2) have a dismal outcome. Furthermore, the combination of *IGHV* mutational status with data derived from chromosome banding analysis allows to identify a subset of patients characterised by M-IGHV without any CK subtypes with a very indolent disease, 90% alive after 10 years of follow-up, who can achieve log-term remission after a short-course of chemoimmunotherapy.

The availability of BCR- and BCL2-inhibitors, alone or in combination, is able to overcome some of the poor-risk prognostic factors associated with CLL, such as clinical stage, TP53 abnormalities and U-IGHV, and new predictive parameters are now emerging.^1,3^ In recent years, the prognostic and predictive role of CK, defined by the presence of at least three chromosomal lesions, is becoming evident at diagnosis,^4,6^ at disease progression^5^ and in relapsed/refractory patients treated with ibrutinib^11^ or venetoclax.^10^ Although CK is found in 14–35% of CLL depending on the studies,^4,8^ it is a heterogeneous cytogenetic category from a quantitative and qualitative point of view. Data from the literature have documented that patients with +12, +18 and +19 although resembling a CK are characterised by an indolent CLL with peculiar clinical features (i.e. female predominance, young age at diagnosis, etc.);^27^ on the other hand the presence of at least five chromosomal aberrations predicted for a very aggressive clinical course independently of the *IGHV* status and *TP53* lesions.^29^ The number of chromosomal lesions was also assessed in our study population, confirming that patients 5 or more aberrations had the shortest TTFT and OS (Fig. S5). Recently, our collaborative group has demonstrated that almost 70% of CK cases harboured major structural aberrations (such as unbalanced translocations, ring or marker chromosomes). This subset, herein called CK2, was associated with a higher incidence of *TP53* aberrations, chemo-refractoriness and a shorter OS at multivariate analysis.^12^ Furthermore, CK2 CLL cases have a distinct mRNA expression profile with a deregulation of genes involved in cell-cycle control and DNA damage response.^12^ In the present study we included 522 patients and we combined data derived from stimulated chromosome banding analysis with the IGHV mutational status in order to improve the prognostic and predictive power of these markers. We demonstrated that M-IGHV patients without any CK subtypes at diagnosis, corresponding to 45% of our cohort, are characterised by a very indolent disease with a median TTFT of 19 years and more 90% of them still alive after 10 years from diagnosis. Although patients with CK2 was a relatively rare subgroup, representing 13% of the cases, most of them (81%) required a treatment within 5 years from the diagnosis, almost all needed a second line of treatment after 3 years from chemoimmunotherapy and the median OS was 7 years.

Although the exact mechanisms which favour the development of a CK are unknown, the strong association between CK and TP53 disruption herein and by other authors^8,28,29^ could play a relevant role. Patients with TP53 mutations are characterised by short telomers and high hTERT expression, a condition known to cause chromosome instability.^30,31^ Patients with TP53 disrupted showed telomere deletion and chromosomal end-to-end fusion in cells with CK.^30^ Thomay et al.^32^ reported that loss or mutation of *TP53* caused an increased number of chromosomal break events leading to dicentric chromosome and whole-arm translocation. In addition, a recent paper from the German CLL Study Group found an association between short telomere length, TP53 abnormalities, early relapse after chemoimmunotherapy and adverse survival.^33^ In particular, cases with 17p- or *TP53* mutations had the shortest telomeres length, increase genomic complexity as well as clonal evolution.^33^

The challenge of contemporary CLL treatment involves attempts to tailor therapy according to the patients’ specific biological risk profile. To responsibly and effectively advance the development of new targeted therapies, novel drugs should be specifically offered to patient subgroups who can gain the greatest benefit compared with established chemoimmunotherapy strategies. Rossi D^34^ et al. demonstrated that OS of M-IGHV patients without 11q or 17p deletions is superimposable to that of the age-matched general population, while U-IGHV subjects and those with high-risk FISH aberrations (i.e. 11q− or 17p−) invariable relapsed after FCR. This observation has been also confirmed in the re-analysis of pivotal clinical trials from the German CLL study group and MD Cancer institute.^35,36^ Gentile M^37^ et al. published a multicentre retrospective study on BR in treatment-naive patients showing that, also with this chemoimmunotherapy, M-IGHV CLL without 11q/17p deletions experienced the best disease control and OS. All these observations clearly identified CLL patients with a low-risk biological profile who can achieved excellent long-term results and disease control with six cycles of chemoimmunotherapy. Our data confirm the above result^34–36^ and extend the observation supporting the notion that front-line chemoimmunotherapy, FCR or BR, represents a highly effective treatment option for physically fit M-IGHV CLL patients without a CK.

Given the disappointing results of CK2 patients with chemoimmunotherapy, we hypothesised that new agents, ibrutinib, would improve the outcome of this unfavourable subset. We compared CK2 patients treated with FCR or BR and those who received ibrutinib as first-line therapy. Although we found some differences between TTNT and OS curves, these were not statistically significant, likely related to the short follow-up (14 months for ibrutinib-treated patients) and the low number of patients (seven CK2 cases treated with ibrutinib). The best first and subsequent therapies for patients with CK are still matter of debate. While the presence of CK has been associated to early relapse in relapsed/refractory patients treated with ibrutinib or venetoclax,^10,11^ the activity of ibrutinib in treatment-naive subjects with a CK has so far not been reported. Overall the literature and current data support the importance of evaluating IGHV mutational status accordingly to recently updated iwCLL guidelines, and suggest that the outcome of CK patients with chemoimmunotherapy is disappointing due to a high rate of chemo-refractoriness, early relapse and short survival.^5,9^

## Conclusions

Thanks to stimulated cytogenetic analysis we identified a CK in 19% of 522 CLL patients. We herein suggest that the combination of IGHV mutation and data derived from chromosome banding analysis allows to refine the prognostic stratification of CLL, to identify M-IGHV patients without any CK subtypes who are characterised by an indolent disease and excellent outcome after chemoimmunotherapy. At the other end, CK2 patients are enriched in cases with *TP53* abnormalities, have unsatisfactory responses to chemotherapy and aggressive diseases with only 40% alive after 10 years of follow-up. New clinical trials incorporating a combination, or a sequence of novel agents should be envisaged for patients with CK, in particular with the CK2 subtype.

## Supplementary information

Supplmentary data and figures

## Data Availability

The datasets generated and analysed during the current study are not publicly available due to the data protection and lack of consent from the patients. Access to data is strictly limited to the researchers who have obtained permission for data processing.
