# Supplementary material for: The combination of complex karyotype subtypes and IGHV mutational status identifies new prognostic and predictive groups in chronic lymphocytic leukaemia
Source: Br J Cancer. 2019 Jun 18;121(2):150–6. doi: 10.1038/s41416-019-0502-x (PMC6738078; doi:10.1038/s41416-019-0502-x)
Supplement: Supplementary file 1 — Supplmentary data and figures [file 41416_2019_502_MOESM1_ESM.docx]

**THE COMBINATION OF COMPLEX KARYOTYPE SUBTYPES AND IGHV MUTATIONAL STATUS IDENTIFIES NEW PROGNOSTIC AND PREDICTIVE GROUPS IN CHRONIC LYMPHOCYTIC LEUKEMIA**

Visentin A, MD^1,2^, Bonaldi L, MD^3^, Rigolin GM, MD, PhD^4^, Mauro FR, MD^5^, Martines A, PhD^3^, Frezzato F, PhD^1,2^, Imbergamo S, MD^1,2^, Scomazzon E, MD^1,2^, Pravato S, MD^1,2^, Bardi, MA PhD^4^, Cavallari M, MD^4^, Volta E, MD^4^, Cavazzini F, MD PhD^4^, Nanni M, PhD^5^, Del Giudice I, MD PhD^5^, Facco M, MS^1,2^, Guarini A, PhD^5^, Semenzato G, MD^1,2^, Foà R, MD^5^, Cuneo A, MD, PhD^4^, Trentin L, MD^1,2,+^.

*1 Hematology and Clinical Immunology Unit, Department of Medicine, University of Padua, Padua, Italy. 2 Venetian Institute of Molecular Medicine, Padua, Italy. 3 Immunology and Molecular Oncology Unit, Veneto Institute of Oncology IOV-IRCSS, Padua, Italy. 4 Hematology section, Department of Medical Sciences, Azienda Ospedaliera-Universitaria, Arcispedale S. Anna, University of Ferrara, Ferrara. 5 Hematology division, Department of Translational and Precision Medicine, "Sapienza" University, Rome, Italy.*

**SUPPLEMENTARY FILES**

Legends to figures pag. 2

Karyotypes of patients with CK pag. 3

Supplementary table S1 pag. 4

Supplementary table S2 pag. 4

Supplementary table S3 pag. 4

Supplementary table S4 pag. 5

Figure S1 pag. 6

Figure S2 pag. 7

Figure S3 pag. 8

Figure S4 pag. 9

Figure S5 pag. 10

**LEGENDS TO FIGURES**

**Figure S1. Kaplan-Meier curves according to IGHV mutational status, presence of complex karyotype and subset of complex karyotype**. In the upper part of the figure we report Kaplan-Meier curves for time to first treatment according to IGHV mutational status (A, mutated vs unmutated IGHV genes), complex karyotype (B, CK vs no CK) and CK subtypes (C, type 2 CK vs type 1 CK vs no CK). In the upper part of the figure Kaplan-Meier curves for overall survival according to IGHV mutational status (D, mutated vs unmutated IGHV genes), complex karyotype (E, CK vs no CK) and the combination of CK subtypes and IGHV status (F, type-2 CK vs type-1 CK vs no CK) are reported. In the bottom (G) part of the figure we compared survival curves according to IGHV mutational status and CK subtypes. The survival curves of patients with mutated-IGHV and unmutated-IGHV genes are superimposable to the one of subjects without CK (p=0.1083) and with type-1 CK (p=0.7988), respectively). Patients with type 2 CK showed the worst prognosis.

**Figure S2. Kaplan-Meier curves of CK2 according to IGHV mutational status and TP53 abnormalities**. In upper panels we report time to first treatment (A) and overall survival (B) of CK2 patients according to the presence of TP53 abnormalities (TP53 abn, including deletions and mutations) or not (TP53 wild type). In lower panels we report time to first treatment (C) and overall survival (D) of CK2 patients according to the IGHV mutational status (M-IGHV, mutated, or U-IGHV, unmutated).

**Figure S3. Kaplan-Meier curves for time to first treated and overall survival according to different prognostic models.** Panels A and B show time to first treatment and overall survival curves according to Dohner’s hierarchical model applied to our cohort of patients. Panels C and D shown time to first treatment and overall survival curves according to Barcelona-Brno score that combined data of IGHV mutational status with the presence of high-risk FISH (i.e. 11q or 17p deletions). Panels E and F shown time to first treatment and overall survival curves according to CLL-IPI score. In the bottom panels we evaluated time to next treatment (i.e. all second line therapy) (G) and overall survival (H) after first line treatment according to the combination of CK subtype with IGHV status. Patients with type 2 CK showed the shorter time to next treatment and survival as compared with U-CK1 (unmutated IGHV and/or CK1) and M-noCK (mutated IGHV without CK).

**Figure S4. Kaplan-Meier curves or CK2 patients**. Time to next treatment (A) and overall survival (B) after start of first line therapy among CK2 patients treated with ibrutinib or chemoimmunotherapy (CIT) FCR or BR.

**Figure S5. Prognostic impact of the number of chromosomal abnormalities.** Time to first treatment (A) and overall survival (B) after diagnosis according to the number of chromosomal abnormalities. Patients with 5 or more aberrations had the shortest time to first treatment and overall survival.

**TABLES**

Table S1. Univariate and multivariate analysis for time to first treatment

|  | **UNIVARIATE ANALYSIS** | | | **MULTIVARIATE ANALYSIS** | | |
| --- | --- | --- | --- | --- | --- | --- |
|  | **HR** | **95% C.I** | **p values** | **HR** | **95% C.I** | **p values** |
| **MALE** | 1.03 | 0.79-1.34 | 0.8298 | - | - | - |
| **AGE>65yy** | 1.03 | 0.79-1.34 | 0.7949 | - | - | - |
| **Binet B-C** | 3.75 | 2.64-5.32 | <0.0001 | 3.98 | 3.05-5.16 | <0.0001 |
| **CD38>30%** | 1.75 | 1.27-2.40 | <0.0001 | 1.94 | 1.46-2.58 | <0.0001 |
| **U-IGHV** | 3.93 | 2.97-5.20 | <0.0001 | 4.20 | 3.15-5.61 | <0.0001 |
| **11q-** | 2.78 | 1.64-4.71 | <0.0001 | 2.83 | 1.98-4.03 | <0.0001 |
| **17p-** | 2.01 | 1.15-3.49 | 0.0008 | 2.01 | 1.33-3.05 | 0.0010 |
| **TP53 abn** | 1.97 | 1.23-3.17 | 0.0002 | 1.98 | 1.37-2.85 | 0.0003 |

Table S2. Univariate and multivariate analysis for overall survival

|  | **UNIVARIATE ANALYSIS** | | | **MULTIVARIATE ANALYSIS** | | |
| --- | --- | --- | --- | --- | --- | --- |
|  | **HR** | **95% C.I** | **p values** | **HR** | **95% C.I** | **p values** |
| **MALE** | 1.35 | 0.86-2.13 | 0.2070 | - | - | - |
| **AGE>65yy** | 4.49 | 2.83-7.13 | <0.0001 | 5.05 | 3.06-8.34 | <0.0001 |
| **Binet B-C** | 2.00 | 1.19-3.36 | 0.0022 | 2.00 | 1.27-3.15 | 0.0027 |
| **CD38>30%** | 2.19 | 1.27-3.77 | 0.0005 | 2.49 | 1.56-3.97 | 0.0001 |
| **U-IGHV** | 2.91 | 1.81-4.69 | <0.0001 | 2.94 | 1.78-4.85 | <0.0001 |
| **11q-** | 2.82 | 1.27-6.29 | 0.0001 | 2.84 | 1.63-4.93 | 0.0002 |
| **17p-** | 2.70 | 1.12-6.46 | 0.0007 | 2.69 | 1.48-4.88 | 0.0011 |
| **TP53 abn** | 1.86 | 0.88-3.93 | 0.0378 | 1.86 | 1.03-3.28 | 0.0408 |

Table S3. Univariate and multivariate analysis for time to next treatment after FCR or BR

|  | **UNIVARIATE ANALYSIS** | | | **MULTIVARIATE ANALYSIS** | | |
| --- | --- | --- | --- | --- | --- | --- |
|  | **HR** | **95% C.I** | **p values** | **HR** | **95% C.I** | **p values** |
| **MALE** | 2.18 | 1.01-4.72 | 0.0473 | 2.93 | 1.03-8.33 | 0.0435 |
| **AGE>65yy** | 2.75 | 1.23-6.11 | 0.0036 | 3.49 | 1.58-7.73 | 0.0020 |
| **Binet B-C** | 1.49 | 0.72-3.09 | 0.1808 | - | - | - |
| **CD38>30%** | 1.24 | 0.59-1.24 | 0.1552 | - | - | - |
| **U-IGHV** | 2.35 | 1.08-5.13 | 0.0316 | 2.24 | 0.78-6.42 | 0.1340 |
| **11q-** | 2.14 | 0.77-5.94 | 0.1459 | - | - | - |
| **17p-** | 5.38 | 1.06-7.24 | 0.0419 | 2.00 | 0.91-6.58 | 0.0640 |
| **TP53 abn** | 5.75 | 1.66-8.50 | 0.0093 | 4.47 | 0.86-7.05 | 0.0418 |
| **M-noCK** | 1.00 | - | - | 1.00 | - | - |
| **U-CK1** | 2.84 | 1.09-7.39 | 0.0323 | 4.28 | 1.03-10.73 | 0.0496 |
| **CK2** | 7.27 | 3.52-9.12 | 0.0004 | 6.74 | 2.40-2.16 | 0.0055 |

Table S4. Univariate and multivariate analysis for overall survival after FCR or BR

|  | **UNIVARIATE ANALYSIS** | | | **MULTIVARIATE ANALYSIS** | | |
| --- | --- | --- | --- | --- | --- | --- |
|  | **HR** | **95% C.I** | **p values** | **HR** | **95% C.I** | **p values** |
| **MALE** | 1.16 | 0.34-4.02 | 0.8212 | - | - | - |
| **AGE>65yy** | 4.54 | 2.94-8.27 | 0.0006 | 3.95 | 2.17-6.38 | 0.0028 |
| **Binet B-C** | 2.47 | 0.83-6.35 | 0.1097 | - | - | - |
| **CD38>30%** | 3.97 | 1.27-7.93 | 0.0230 | 3.11 | 1.20-6.81 | 0.0391 |
| **U-IGHV** | 2.18 | 1.03-9.27 | 0.0289 | 1.97 | 0.98-5.92 | 0.0704 |
| **11q-** | 4.11 | 1.81-7.96 | 0.0297 | 3.73 | 2.14-7.19 | 0.0317 |
| **17p-** | 3.70 | 1.28-8.35 | 0.0316 | 3.19 | 0.89-7.73 | 0.0619 |
| **TP53 abn** | 3.15 | 1.56-8.12 | 0.0391 | 2.79 | 1.06-6.52 | 0.0475 |
| **M-noCK** | 1.00 | - | - | 1.00 | - | - |
| **U-CK1** | 3.36 | 0.62-8.12 | 0.1581 | 2.37 | 0.29-9.04 | 0.4160 |
| **CK2** | 6.19 | 2.25-10.70 | 0.0065 | 5.23 | 1.65-8.66 | 0.0113 |

Karyotypes of patients classified as harboring a complex karyotype.
